# TARBP2-Enhanced Resistance during Tamoxifen Treatment in Breast Cancer

**DOI:** 10.3390/cancers11020210

**Published:** 2019-02-12

**Authors:** Ming-Yang Wang, Hsin-Yi Huang, Yao-Lung Kuo, Chiao Lo, Hung-Yu Sun, Yu-Jhen Lyu, Bo-Rong Chen, Jie-Ning Li, Pai-Sheng Chen

**Affiliations:** 1 Department of Surgery, National Taiwan University Hospital, Taipei 100, Taiwan; Joylo312@ms14.hinet.net (C.L.); sallyliu96@hotmail.com (Y.-J.L.); yudawn@gmail.com (B.-R.C.); 2 Department of Pathology, National Taiwan University Hospital, Taipei 100, Taiwan; hyhuang4010@gmail.com; 3 Department of Surgery, National Cheng Kung University Hospital, College of Medicine, National Cheng Kung University, Tainan and Dou-Liou Branch, Tainan 704, Taiwan; YLKUO@mail.ncku.edu.tw; 4 Department of Biomedical Engineering, College of Biology, Hunan University, Changsha 410006, China; s5893149@hnu.edu.cn; 5 Department of Medical Laboratory Science and Biotechnology, College of Medicine, National Cheng Kung University, Tainan 704, Taiwan; jessyli61621@gmail.com; 6 Institute of Basic Medical Sciences, College of Medicine, National Cheng Kung University, Tainan 704, Taiwan

**Keywords:** tamoxifen, hormone therapy, TARBP2, SOX2, merlin

## Abstract

Tamoxifen is the most widely used hormone therapy in estrogen receptor-positive (ER+) breast cancer, which accounts for approximately 70% of all breast cancers. Although patients who receive tamoxifen therapy benefit with respect to an improved overall prognosis, resistance and cancer recurrence still occur and remain important clinical challenges. A recent study identified TAR (HIV-1) RNA binding protein 2 (TARBP2) as an oncogene that promotes breast cancer metastasis. In this study, we showed that TARBP2 is overexpressed in hormone therapy-resistant cells and breast cancer tissues, where it enhances tamoxifen resistance. Tamoxifen-induced TARBP2 expression results in the desensitization of ER+ breast cancer cells. Mechanistically, tamoxifen post-transcriptionally stabilizes TARBP2 protein through the downregulation of Merlin, a TARBP2-interacting protein known to enhance its proteasomal degradation. Tamoxifen-induced TARBP2 further stabilizes SOX2 protein to enhance desensitization of breast cancer cells to tamoxifen, while similar to TARBP2, its induction in cancer cells was also observed in metastatic tumor cells. Our results indicate that the TARBP2-SOX2 pathway is upregulated by tamoxifen-mediated Merlin downregulation, which subsequently induces tamoxifen resistance in ER+ breast cancer.

## 1. Introduction

Breast cancer is the most common malignancy in women worldwide and treatment failure remains a major challenge. According to the World Health Organization (WHO), more than 2 million new cases are diagnosed, and more than 600,000 women die from breast cancer each year [1]. Based on the expression of estrogen receptor (ER) in tumor tissues, approximately 70% of breast cancers are ER-positive (ER+). ER alpha (ERα) was first identified in 1958, and its expression was later found in breast, endometrial, and ovarian tissues. Theoretically, activation of the ER signaling pathway facilitates proliferation and tumorigenesis of breast cancer cells, and thus hormone therapy is the major treatment for ER+ breast cancer patients. As a hormone receptor, ERα is activated by estrogen, after which it translocates into the cell nucleus where it can bind to specific regions of DNA to regulate gene expression [2]. Activation of the ER signaling pathway promotes proliferation and tumorigenesis of breast cancer cells [3]. Tamoxifen, which is a selective estrogen-receptor modulator (SERM) that was discovered in 1967, has been the gold standard first-line hormonal therapy for ER+ breast cancer for more than 45 years [4]. Currently, tamoxifen is widely used to treat all stages of breast cancer and for chemoprevention in women at high risk for breast cancer. It has also been used for the improvement of bone mineral density in postmenopausal women. Despite the fact that ER+ breast cancer exhibits a high initial response to hormonal therapy, drug resistance and cancer recurrence ultimately develop [4,5,6], especially in metastatic breast cancer patients who are treated with tamoxifen [7,8,9]. At the molecular level, several mechanisms are responsible for tamoxifen resistance: overexpression/activation of the coactivator of ER, downregulation/inhibition of the corepressor of ER, and active mutations or ligand-independent activation of ER. In addition, activation of growth factor receptors, such as EGFR and HER2, has been identified in tamoxifen-resistant cells to induce the MAPK and PI3K/Akt signaling pathways and to enhance mitogenic and antiapoptotic effects. This provides resistant cells with a compensatory survival skill that is independent of the ER pathway [10,11].

TARBP2 (TAR (HIV-1) RNA binding protein 2) is an RNA binding protein that exhibits several known functions [12]. At the molecular level, TARBP2 suppresses the activation of interferon (IFN)-induced dsRNA-regulated protein kinase PKR and interacts with the PKR activator PACT [13]. TARBP2 also regulates HIV-1 gene expression through its interaction with TAR [14] and is also involved in the RNAi/miRNA pathway as a cofactor that binds to Dicer in the RISC complex [15]. Biologically, the role of TARBP2 in development was observed in TARBP2 knockout mice, which exhibit growth defects [16]. The expression of TARBP2 enhances a transformed phenotype and tumorigenesis in vivo [17]. The oncogenic function of TARBP2 in breast cancer was unclear until Goodarzi et al. published a research demonstrating that TARBP2 enhances the metastasis of breast cancer cells [18]. Goodarzi et al. revealed that through binding and destabilizing the metastatic suppressors APP and ZNF395, the elevated level of TARBP2 in breast cancer cells enhances distant metastasis [18]. In this study, we found that the expression of TARBP2 was dramatically upregulated in tamoxifen-resistant cells. Moreover, the induction of TARBP2 was found to be directly triggered by tamoxifen treatment, which suggests a therapy-induced drug resistance pathway that should be considered when tamoxifen is used, as it provides crucial information for the development of possible therapeutic strategies. 

## 2. Materials and Methods

### 2.1. Cell Culture

MCF-7 cells were cultured in low glucose Dulbecco’s modified Eagle medium (DMEM) supplemented with 10% fetal bovine serum (FBS), 2% glutamine and 1% penicillin/streptomycin antibiotics. DMEM powder with low glucose and RPMI-1640 were purchased from HiMedia (Mumbai, India). FBS was purchased from Corning (New York, NY, USA). Glutamine and penicillin/streptomycin antibiotics were purchased from HiMedia. Tamoxifen-resistance cell lines (TR1, TR2, TR3) were established by culturing parental MCF-7 cells in the presence of 3 μM tamoxifen over a period of 6 months. Tamoxifen was purchased from Sigma (Darmstadt, Germany). ZR-75-75-1 cells were maintained in RPMI-1640 medium supplemented with 10% fetal bovine serum (FBS), 2% glutamine and 1% penicillin/streptomycin antibiotics. All cells were incubated at 37 °C with 5% CO_2_ in a humidified incubator.

### 2.2. Western Blot

Short hairpin-constructed plasmids for lentivirus production were purchased from the National RNAi Core Facility Platform located at the Institute of Molecular Biology/Genomic Research Center, Academia Sinica, Taipei, Taiwan. Plasmid was transfected into cells for 24 h by using the HyFectTM DNA transfection reagent according to the manufacturer’s protocol (Leadgene Biomedical, Tainan, Taiwan). Whole cell extracts were lysed in RIPA buffer and collected to fractionate by SDS-PAGE, and then transferred onto PVDF membranes according to the manufacture’s protocols (Bio-Rad, Hercules, CA, USA). PVDF membrane was purchased from GE Healthcare (Uppsala, Sweden). After blocking with 5% nonfat milk in TBST for 60 min, membranes were washed and incubated with primary antibodies at 4 °C overnight. Membranes were washed three times for 10 min and incubated with secondary antibodies for 60 min. Protein expressions were visualized by ECL system according to the manufacture’s protocols. ECL (Enhanced Chemiluminescent) was purchased from PerkinElmer (Waltham, MA, USA).

### 2.3. RNA Extraction and Reverse Transcription Real-Time PCR

Total RNA was isolated by using Trizol reagent according to the manufacturer’s instructions. Trizol reagent was purchased from Invitrogen (Waltham, MA, USA). 200 ng of RNA was reverse transcribed to complementary DNA (cDNA) by using a reverse transcriptase enzyme, a random primer, dNTPs and an RNase inhibitor. Revert Aid First Strand cDNA Synthesis Kit was purchased from Thermo Fisher (Waltham, MA, USA). Real-time PCR was performed by Applied Biosystem Step One Real-time PCR system (Applied Biosystems, CA, USA) according to manufacturer’s protocols. Samples run in three independent experiments and GAPDH (Glyceraldehyde-3-Phosphate Dehydrogenase) or α-tubulin was used as an internal control to normalize the target genes.

### 2.4. MTT Assay

Cell viability was analyzed by MTT (3,(4,5-dimethylthiazol-2-yl-) diphenyltetrazolium bromide, 5 mg/mL). MTT powder and DMSO was purchased from Cyrusbioscience (Taipei, Taiwan). Cells were seeding in 96-well plates and incubated overnight, and then changed into the fresh medium containing indicated concentrations of tamoxifen for 72 h 50 μL of MTT was added to each well and incubated for 2 h, and the purple formazan crystals were dissolved in DMSO (Dimethyl sulfoxide). The absorbance was measured by a microplate reader at a wavelength of 570 nm.

### 2.5. Colony Formation Assay

0.5 × 10^3^ cells were seeded on the 6-well plates and incubated overnight, and 2 μM of tamoxifen was added to the medium for 7 days. The cells were fixed by 3.7% formaldehyde and stained by 0.05% crystal violet. Crystal violet purchased from Sigma.

### 2.6. Patients and Specimens

Breast cancer patients had operation for breast cancer and subsequently developed lymph node metastasis at National Taiwan University Hospital were included during 2011 to 2015. Paraffin-embedded, formalin-fixed surgical resection specimens were collected for immunohistochemical staining for TARBP2 and SOX2. All these patients had ER positive disease and had anti-hormone therapy for their disease. Tumor size, local invasion, and lymph node metastasis were acquired from pathology reports. Breast cancer tissues obtained from NTU Hospital were used according to IRB protocols approved by the IRB committee of NTU Hospital (NTUH-REC No.: 201612165RIND). The protocol and the request for the waiver of informed consent for use of existing biosamples have been approved by the Research Ethics Committee D of the NTU Hospital.

### 2.7. Immunohistochemistry

Immunohistochemical studies were performed on formalin-fixed, paraffin-embedded tissue. Tissue sections were deparaffinized according to established procedures. Antigen retrieval was performed at pH 9.0 using Epitope Retrieval 2 solution (Leica Microsystems, Wentzlar, Germany) for 20 min at 100 °C. The primary antibodies used were anti-SOX2 (Millipore, MA, USA; cat. AB5603, 1:50) anti-TARBP2 (Thermo, MA, USA; cat. LF-MA0209, Clone 46D1, 1:600) for 30 min. Slides were then stained using the Leica Microsystems BONDMAX autostainer according to the following steps. Post primary IgG linker reagent localized mouse antibody for 8 min. Poly-HRP IgG reagent localized rabbit antibody for 8 min. Staining was developed with the substrate chromogen, DAB for 10 min. The sections were counterstained with modified Mayer’s hematoxylin for 5 min. The staining intensity was evaluated by pathologist.

### 2.8. Statistical Analysis

All experiments were performed as the means ± SEM. The statistical significance between different groups was analyzed by one-way or two-way ANOVA using the Prism 7 software (GraphPad Software, San Diego, CA, USA). Values were considered significant when *p* value was less than 0.05.

## 3. Results

### 3.1. TARBP2 Is Overexpressed in Hormone Therapy-Resistant Cells and Breast Cancer Tissues

**Figure 1:**
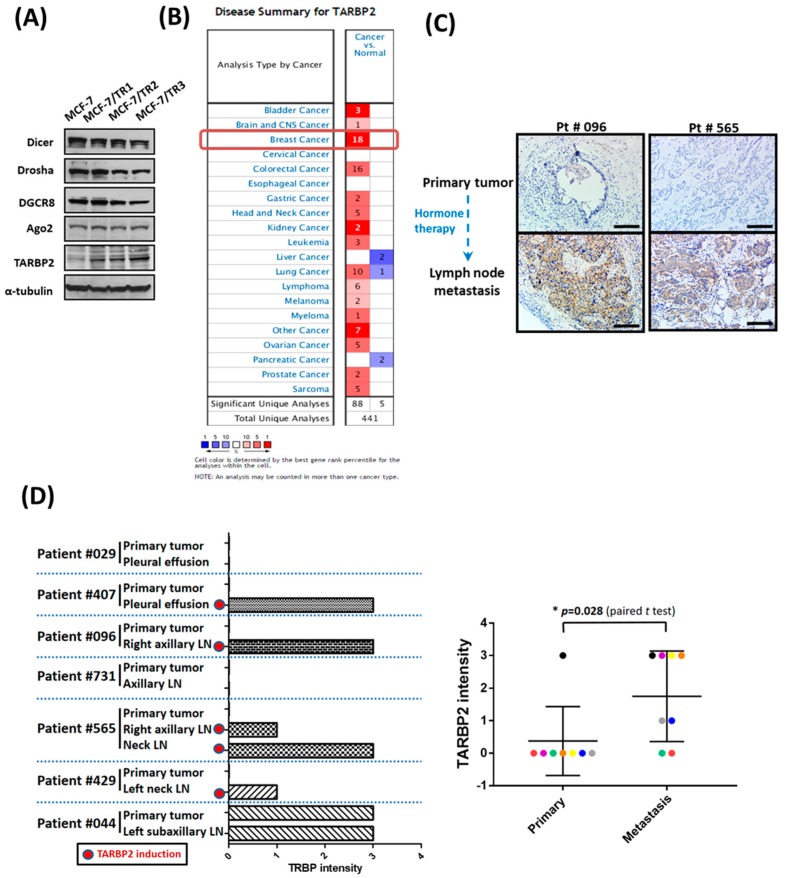
TARBP2 is overexpressed in hormone therapy resistant cells and breast cancer tissues. (**A**) Screening for the expression of different microRNA biogenesis factors in tamoxifen-sensitive cells (MCF-7) and tamoxifen-resistant cells (TR1, TR2, TR3). Cells were seeded in the plates and cultured until they reached 70–80% confluence; they were then collected to analyze the expression of TARBP2 by western blot. (**B**) The expression of TARBP2 was analyzed and downloaded using Oncomine (www.oncomine.org). Re-used from [22] (**C**,**D**) Association of TARBP2 expression and hormone therapy resistance in breast cancer tissues. Representative images of TARBP2 IHC in primary tumors and tumors in lymph nodes in cases of cancer recurrence (**C**). Scale Bar: 100 uM. Statistics of TARBP2 protein expression levels in primary tumors and metastatic tumor cells in in cases of cancer recurrence (**D**).

The dysregulation of miRNA and protein factors that are involved in miRNA biogenesis has been reported in human malignancies [19,20,21]; however, the roles of these factors in hormone therapy resistance remain unclear. To determine the expression level of these proteins, we established tamoxifen-resistant MCF-7 cells (TR1, TR2, TR3) and confirmed the resistance of these cells (Supplementary Figure S1A,B). After screening for the expression of miRNA biogenesis factors, we found that only TARBP2 expression was upregulated in tamoxifen-resistant cells (Figure 1A). Interestingly, we also found that TARBP2 expression was significantly upregulated in breast cancer compared with normal tissues in all datasets (18/18; 100%) in the Oncomine database (Figure 1B). Also, elevated TARBP2 level was observed in different subtypes of breast cancer (Supplementary Figure S2A). Furthermore, in ER+ patients (Supplementary Figure S2B) and ER+ patients treated with adjuvant tamoxifen therapy (Figure S2C,D), higher TARBP2 expression was observed to be significantly correlated with poor prognosis. To establish whether the upregulation of TARBP2 in tamoxifen-resistant breast cancer cells could be observed in human tumors, we collected metastatic tumors and their corresponding primary tumors from breast cancer patients receiving hormone therapy and analyzed TARBP2 expression in these tissues by IHC (Figure 1C,D). Consistent with our in vitro findings, TARBP2 was highly expressed in tumor cells in metastatic lymph nodes or pleural effusions compared with paired primary tumors from the same patient (Figure 1D). In seven paired tissues, a higher level of TARBP2 protein was observed in five metastatic sites from breast cancer patients (Figure 1D). These results indicated that an elevated TARBP2 level is correlated with poor prognosis of ER+ patients and is associated with enhanced tamoxifen resistance.

### 3.2. Elevated TARBP2 Promotes Acquired Resistance to Tamoxifen

**Figure 2:**
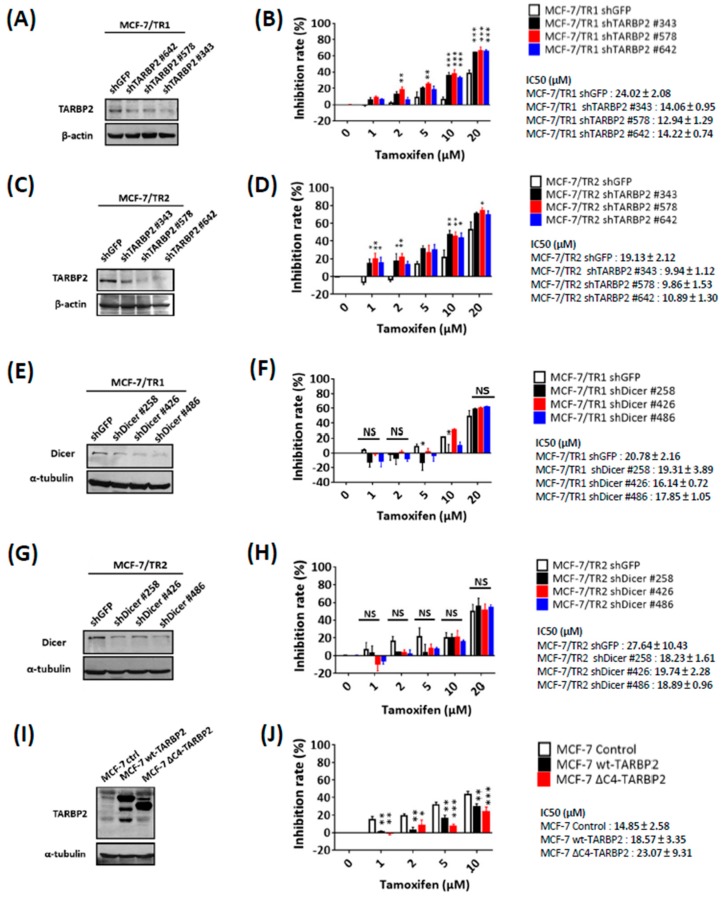
TARBP2 confers tamoxifen resistance in breast cancer cells through a Dicer-independent pathway. (**A**–**D**) Effect of TARBP2 in tamoxifen resistance. MCF-7/TR1 (**A**) and TR2 (**C**) cells were transfected with the indicated shRNA targeting TARBP2 for 48 h, and the efficiency of TARBP2 knock-down was examined by western blot. Cells transfected with the indicated shRNA were treated with different concentrations of tamoxifen (1, 2, 5, 10, 20 μM) for 72 h, and cell proliferation was determined by MTT assay (**B**,**D**). (**E**–**H**) Effect of Dicer on TR1 and TR2 cells in response to tamoxifen. MCF-7/TR1 (**E**) and TR2 (**G**) cells were transfected with the indicated shRNAs targeting Dicer for 48 h, and the efficiency of Dicer knock-down was examined by western blot. Cells transfected with the indicated shRNA were treated with different concentrations of tamoxifen (1, 2, 5, 10, 20 μM) for 72 h, and cell proliferation was determined by MTT assay (**F**,**H**). (**I**,**J**) Effects of microRNA-independent functions of TARBP2 on tamoxifen sensitivity. Cells were transfected with either the control or wt-TARBP2, ΔC4-TARBP2 plasmid. After 24 h of incubation, the cells were harvested to determine the TARBP2 expression by western blot (**I**). Cells as indicated in (**I**) were treated with different concentrations of tamoxifen (1, 2, 5, 10, 20 μM) for 72 h, after which an MTT assay was performed to evaluate cell viability. All results of MTT cell proliferation assay results are presented as the means ± SEM from at least three separate experiments that were performed in duplicate or triplicate and analyzed by two-way ANOVA. * *p* ≤ 0.05, ** *p* ≤ 0.01, *** *p* ≤ 0.001. NS: no significance, *p* > 0.05.

To investigate the potential role of TARBP2 in the modulation of tamoxifen resistance, we knocked down TARBP2 in MCF-7/TR1 and MCF-7/TR2 cells using three specific shRNAs (Figure 2A,C). These cells were treated with different doses of tamoxifen and were subjected to MTT assay to evaluate their drug sensitivity (Figure 2B,D). The depletion of TARBP2 significantly enhanced tamoxifen sensitivity of MCF-7/TR1 and MCF-7/TR2 cells (Figure 2B,D), which indicated that TARBP2 upregulation is essential for acquired tamoxifen resistance. Since one of the functions of TARBP2 is to interact with Dicer to modulate miRNA biogenesis [15], we also knocked down Dicer in MCF-7/TR1 and MCF-7/TR2 cells to investigate whether tamoxifen resistance also relies on its function in miRNA regulation (Figure 2E,G). Unlike the knockdown of TARBP2, the knockdown of Dicer did not affect the sensitivity of MCF-7/TR1 and MCF-7/TR2 cells to tamoxifen (Figure 2F,H). Moreover, we transfected the cells with a C4-truncated TARBP2, which has lost its Dicer-binding domain, to further confirm whether TARBP2-enhanced resistance acts through the miRNA pathway (Figure 2I). Consistently, enhanced tamoxifen resistance was observed in MCF-7 cells after TARBP2 overexpression (Figure 2I,J). The promoting effects were also observed in cells that overexpressed C4-truncated TARBP2 (Figure 2I,J). Together, these results indicate that the upregulation of TARBP2 confers acquired resistance to tamoxifen in breast cancer cells.

### 3.3. Tamoxifen-Induced TARBP2 Results in the Desensitization of ER+ Breast Cancer Cells

**Figure 3:**
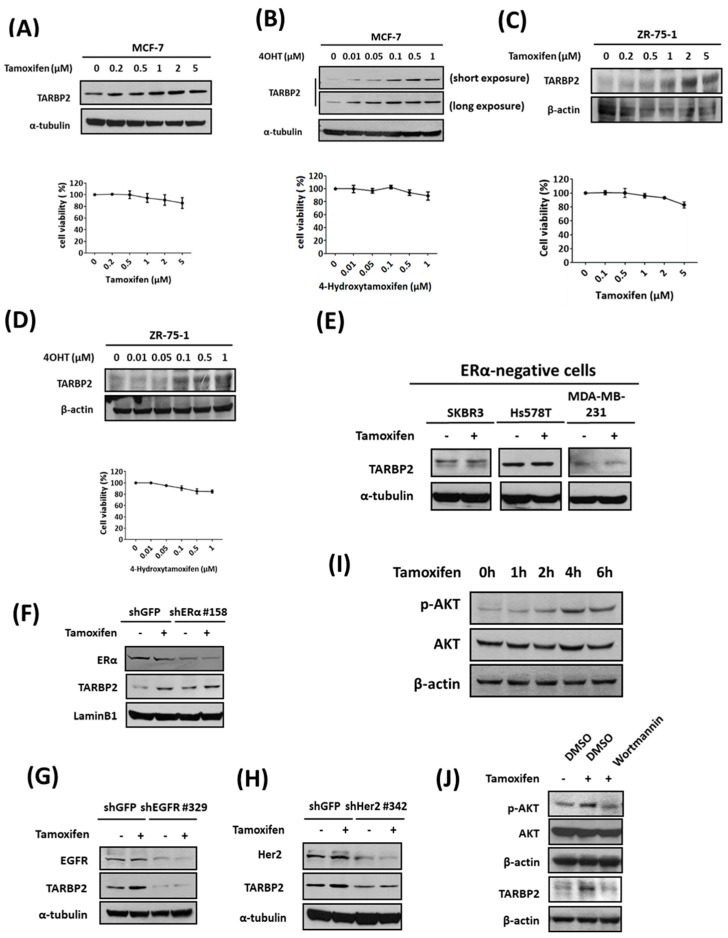
TARBP2 is induced by tamoxifen treatment in ERα-positive breast cancer cells. (**A**–**D**) Association of TARBP2 expression and tamoxifen treatment in ERα-positive breast cancer cells. MCF-7 (**A**,**B**) and ZR-75-1 (**C**,**D**) cells were treated with increasing concentrations of tamoxifen or 4-hydroxytamoxifen for 48 h, and a western blot was performed to examine TARBP2 expression. The cytotoxic effects of the indicated concentrations were evaluated by MTT assay. All MTT results are presented as the means ± SEM from at least three separate experiments that were performed in duplicate or triplicate. (**E**) TARBP2 expression in ER-negative breast cancer cells treated with tamoxifen. ER-negative cells were collected to determine TARBP2 expression by western blot after treatment with tamoxifen for 48 h. (**F**–**H**) MCF-7 cells were introduced with shRNAs targeting ERα (**F**), EGFR (**G**) and Her2 (**H**) for 48 h; 2 μM tamoxifen was then then added to the culture medium for 48 h. The cells were harvested to determine the protein expressions by western blot. (**I**) MCF-7 cells were treated with 2 μM tamoxifen and harvested at the indicated time point to analyze the expressions of p-AKT and AKT by western blot. (**J**) MCF-7 cells were pre-treated 100 nM wortmannin to inhibit AKT phosphorylation for 1 h. After 4 h (p-AKT and AKT) and 48 h (TARBP2) of tamoxifen treatment, the protein expressions were analyzed by western blot.

Drug resistance may arise from the changes in expression that are observed in resistant cells during the selection process and the expansion of cells that survived. In this case, TARBP2 upregulation will be observed only in MCF-7/TR cells that are selected over the long term using tamoxifen. Our previous results showed that TARBP2 is upregulated in tamoxifen-resistant breast cancer cells and tumors and that TARBP2 contributes to acquired resistance. Surprisingly, we observed that the treatment of parental MCF-7 cells with tamoxifen also induced TARBP2 expression in a dose-dependent manner (Figure 3A, left) without the induction of significant cytotoxic effects (Figure 3A, right). Similar phenomena were also observed in MCF-7 cells treated with 4-Hydroxytamoxifen (4OHT), the metabolically active form found in the human body (Figure 3B). To confirm these effects in different ER+ breast cancer cells, we used ZR-75-1 cells for treatment with tamoxifen or 4-OHT and observed similar dose-dependent effects of both drugs on the induction of TARBP2 expression under short-term and noncytotoxic treatments (Figure 3C,D). In contrast, no significant induction of TARBP2 was found in the ER- breast cancer cell lines SKBR3, Hs578T, and MDA-MB-231 (Figure 3E). These results indicate that the induction of TARBP2 is driven by tamoxifen treatment in ER+ breast cancer cells. Moreover, we also addressed whether the tamoxifen-induced TARBP2 acts through ER, EGFR, or Her2; and found that knockdown EGFR and Her2, but not ER, abolish the TARBP2 induction induced by tamoxifen (Figure 3F–H). Since AKT is the downstream of both EGFR and Her2 pathways [23], we determined the change of AKT phosphorylation and observed a significant induction of phosphor-AKT after 4 h of tamoxifen treatment (Figure 3I), while inhibiting AKT by chemical inhibitor diminished the tamoxifen-induced TARBP2 (Figure 3J). These results further support that the TRBP induction is triggered by tamoxifen through an ER-independent pathway.

**Figure 4:**
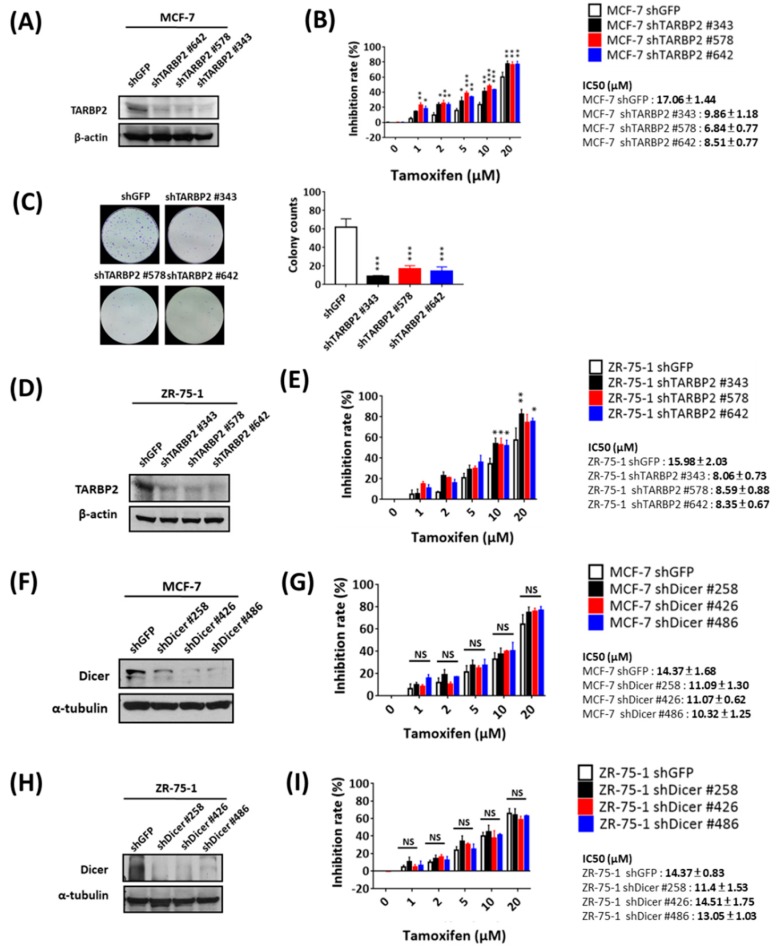
Tamoxifen-induced TARBP2 contributes to acquired resistance to tamoxifen. (**A**–**E**) Effect of TARBP2 on tamoxifen sensitivity in MCF-7 and ZR-75-1 cells. MCF-7 (**A**,**B**) and ZR-75-1 (**D**,**E**) cells were transfected with the indicated shRNAs targeting TARBP2 for 48 h, and the efficiency of TARBP2 knock-down was examined by western blot. Cells with transfected with the indicated shRNA were treated with different concentrations of tamoxifen (1, 2, 5, 10, 20 μM) for 72 h, and the proliferation and colony-forming ability were determined by MTT (**B**,**E**) and colony formation assays (**C**). (**F**–**I**) Effect of Dicer expression on ER+ cells treated with tamoxifen. MCF-7 (**F**) and ZR-75-1 (**H**) cells were transfected with the indicated shRNAs targeting Dicer for 48 h, and the efficiency of Dicer knock-down was examined by western blot. Cells transfected with the indicated shRNA were treated with different concentrations of tamoxifen (1, 2, 5, 10, 20 μM) for 72 h, and cell proliferation was determined by MTT assay (**G**,**I**). The results from all experiments are provided as the means ± SEM from at least three separate experiments that were performed in duplicate or triplicate and analyzed by two-way ANOVA. * *p* ≤ 0.05, ** *p* ≤ 0.01, *** *p* ≤ 0.001.

Considering that TARBP2 is upregulated and involved in the acquired resistance of MCF-7/TR cells, we wondered whether tamoxifen-induced TARBP2 also enhances primary resistance. To investigate the functional contribution of TARBP2, we used shRNAs to block tamoxifen-induced TARBP2 and determined the viability of MCF-7 cells in the presence of tamoxifen (Figure 4A). At different doses of tamoxifen treatment, cells in which TARBP2 was knocked down by specific shRNAs exhibited higher tamoxifen sensitivity according to MTT (Figure 4B) and colony formation (Figure 4C) assays. Similarly, these effects were also observed in tamoxifen-treated ZR-75-1 cells in which TARBP2 was knocked down (Figure 4D,E). These evidences suggesting that the upregulation of TARBP2 is directly triggered by tamoxifen and consequently results in enhanced primary tamoxifen resistance. Again, the knockdown of Dicer in tamoxifen-treated MCF-7 (Figure 4F,G) and ZR-75-1 (Figure 4H,I) cells did not affect drug sensitivity, which supports the concept that tamoxifen-induced TARBP2 enhances drug resistance in a miRNA-independent manner.

### 3.4. Tamoxifen Posttranscriptionally Stabilizes TARBP2 Protein Expression through Downregulation of Merlin

**Figure 5:**
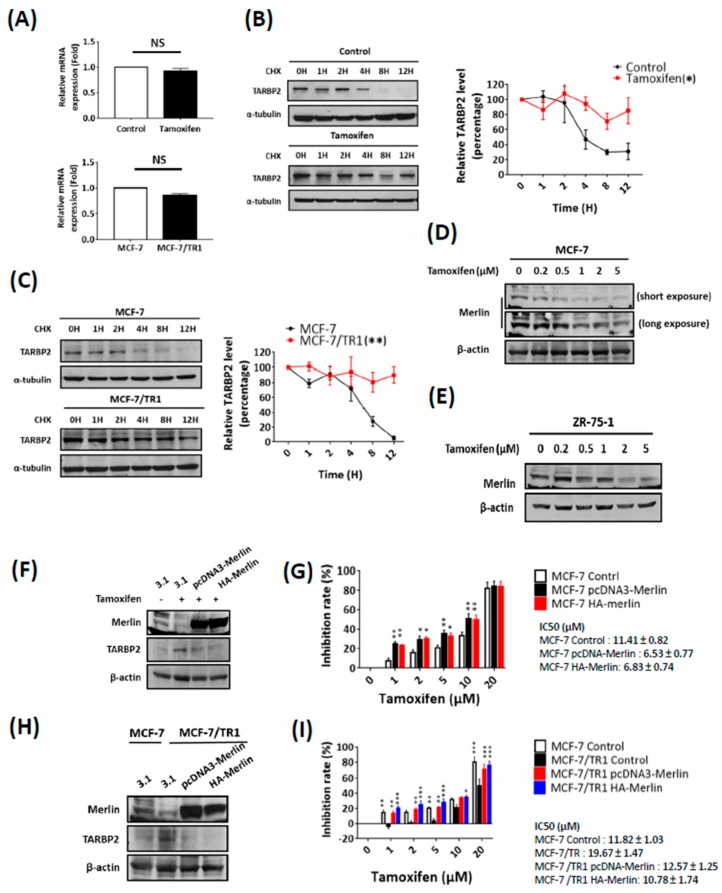
Tamoxifen stabilizes TARBP2 through downregulation of Merlin. (**A**–**C**) Tamoxifen enhanced the protein stability of TARBP2. RNA was isolated from cells (MCF-7 cells were pretreated with 2 μM tamoxifen for 48 h; MCF-7/TR1 cells was seeded in plates and cultured until they reached 70–80% confluence in the presence of tamoxifen) to analyze the mRNA level of TARBP2 by reverse-transcription PCR (qRT-PCR). The experiments were repeated at least 3 times (**A**). Cells as indicated in (**A**) were treated with 50 μg/mL cycloheximide to block protein synthesis and were harvested at the indicated time point to analyze the expression of TARBP2 by western blotting (**B**,**C**). The degradation rates were plotted for the average ± SEM of at least three independent experiments and analyzed by two-way ANOVA. * *p* ≤ 0.05, ** *p* ≤ 0.01. (**D**–**I**) Role of Merlin in tamoxifen sensitivity through regulation of TARBP2 expression. MCF-7 (**D**) and ZR-75-1 (**E**) cells were treated with increasing concentrations of tamoxifen for 48 h, and western blot was performed to detect the expression of TARBP2 and Merlin. MCF-7 and TR1 cells were transfected with the indicated plasmids to overexpress Merlin for 24 h; cells were then collected to analyze the expression of TARBP2 and Merlin (**F**,**H**). Cells as indicated in (**F**,**H**) were treated with different concentrations of tamoxifen (1, 2, 5, 10, 20 μM) for 72 h, and cell proliferation was determined by MTT assay (**G**,**I**). All MTT results are presented as the means ± SEM from at least three separate experiments that were performed in duplicate or triplicate and analyzed by two-way ANOVA. * *p* ≤ 0.05, ** *p* ≤ 0.01, *** *p* ≤ 0.001.

We next sought to determine the mechanism of tamoxifen-mediated TARBP2 induction. First, we analyzed the mRNA expression of TARBP2 and found that the TARBP2 mRNA level was not significantly changed either in tamoxifen-treated MCF-7 cells or in MCF-7/TR1 cells (Figure 5A), which suggested that the regulation may occur at the posttranscriptional level. Following these findings, we further treated these cells with cycloheximide (CHX) to block their de novo protein synthesis in order to investigate changes in TARBP2 protein stability (Figure 5B). Interestingly, a significant prolonged degradation of TARBP2 protein was observed in both tamoxifen-treated MCF-7 cells compared with untreated MCF-7 cells (Figure 5B) and in MCF-7/TR1 cells compared with parental MCF-7 cells (Figure 5C). 

These results indicated that tamoxifen treatment facilitates the accumulation of TARBP2 protein via a decrease in its degradation. Merlin was identified as a TARBP2-interacting protein suppressed by AKT and promotes the ubiquitination and degradation of TARBP2 [24,25,26]. Our experiments also showed that the expression of the Merlin protein was suppressed in a dose-dependent manner in tamoxifen-treated MCF-7 (Figure 5D) and ZR-75-1 (Figure 5E) cells. In addition, the reduced polyubiquitination was also observed in tamoxifen-treated and resistant MCF-7 cells (Figure S3A,B). These results suggested a possible mechanism that tamoxifen inhibits Merlin to suppress TARBP2 protein degradation. Thus, we restored Merlin expression and determined TARBP2 expression in tamoxifen-treated MCF-7 (Figure 5F) and MCF-7/TR1 (Figure 5H) cells. Upon the restoration of Merlin, the upregulated TARBP2 was completely diminished both in tamoxifen-treated (Figure 5F) and in MCF-7/TR1 (Figure 5H) cells. It is also showed that the restoration of Merlin resensitizes tamoxifen-treated MCF-7 (Figure 5G) and MCF-7/TR1 (Figure 5I) cells to tamoxifen. Overall, these results indicate that tamoxifen downregulates Merlin to stabilize the TARBP2 protein, which results in enhanced primary and acquired resistance to tamoxifen.

### 3.5. Tamoxifen-Induced TARBP2 Stabilizes SOX2 Protein to Enhance Desensitization of Breast Cancer Cells to Tamoxifen

**Figure 6:**
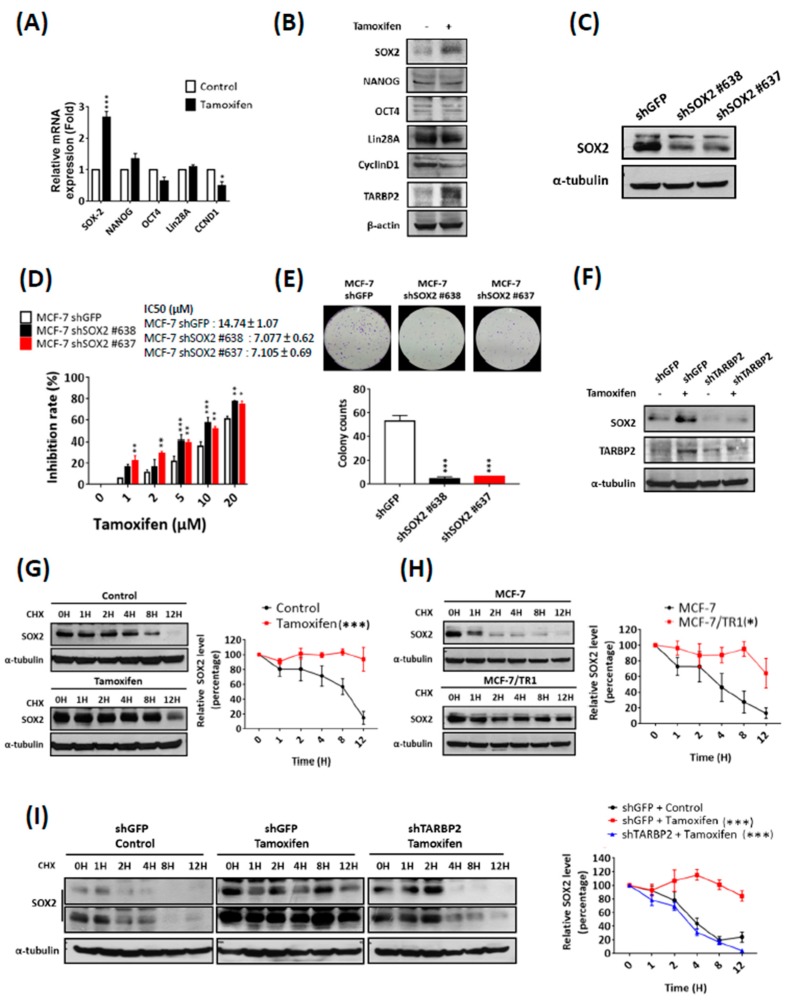
Tamoxifen induces SOX2 to enhance tamoxifen resistance through TARBP2. (**A**,**B**) Expression of different stem cell markers after tamoxifen treatment. MCF-7 cells were treated with 2 μM tamoxifen for 48 h and then RNA was isolated to analyze the mRNA expression of stem cell markers by reverse-transcription PCR (qRT-PCR). The experiments were repeated at least 3 times, and ATP5E was used as a positive control for tamoxifen treatment (**A**). * *p* ≤ 0.05 by *t*-test. Cells as indicated in (**A**) were collected to analyze protein expression by western blotting (**B**). (**C**,**D**) Effect of SOX2 expression on tamoxifen sensitivity. MCF-7 cells were transfected with shRNA targeting SOX2 for 48 h and treated with different concentrations of tamoxifen (1, 2, 5, 10, 20 μM) for 72 h. The efficiency of SOX2 knock-down was examined by western blot (**C**), and the proliferation and colony formation were determined by MTT (**D**) and colony formation assays (**E**), respectively. MTT experimental results are given as the means ± SEM from at least three separate experiments that were performed in duplicate or triplicate and analyzed by two-way ANOVA. * *p* ≤ 0.05, ** *p* ≤ 0.01. (**F**,**G**) Tamoxifen downregulated the protein level of SOX2 through TARBP2. MCF-7 cells were transfected with shRNAs targeting TARBP2 for 48 h; 2 μM tamoxifen was then added to the culture medium for 48 h. The cells were harvested to determine the protein expressions by western blot. (**G**–**I**) TARBP2-regulated protein stability of SOX2 in tamoxifen-treated and resistant cells. Tamoxifen-treated (2 μM for 48 h) MCF-7 (**G**) and MCF-7/TR1 (**H**) cells were treated with 50 μg/mL cycloheximide to block protein synthesis and were then harvested at the indicated time point to analyze the expression of SOX2 by western blotting. (**I**) MCF-7 cells were transfected with the indicated shRNAs targeting TARBP2 for 48 h and treated with 2 μM tamoxifen for 48 h. Cells were add 50 μg/mL cycloheximide and harvested at the indicated time point to analyze the expression of SOX2 by western blotting. The degradation rates were plotted for the average ± SEM of at least three independent experiments and analyzed by two-way ANOVA. * *p* ≤ 0.05, ** *p* ≤ 0.01, *** *p* ≤ 0.001.

To identify the downstream modulator of TARBP2, we determined the expression of several key factors that have been reported to modulate self-renewal and drug resistance of cancer cells, including SOX2, Nanog, OCT4, Lin28A, CCND1 [27,28,29,30]. We used real-time qPCR to screen for the expression of these proteins and found that the expression of SOX2 mRNA was upregulated by tamoxifen treatment (Figure 6A). The upregulated expression of SOX2 protein was further confirmed by western blot (Figure 6B), with the reduction of cyclin D1 which is a known target inhibited by tamoxifen treatment (Figure 6A,B). After the knockdown of SOX2 using specific shRNAs (Figure 6C), the tamoxifen sensitivity of MCF-7 cells was rescued according to MTT (Figure 6D) and colony formation (Figure 6E) assays, which indicates its functional role in the promotion of tamoxifen resistance. We next investigated whether the upregulation of SOX2 is controlled by TARBP2 and found that tamoxifen also enhanced SOX2 expression (Figure 6F). Moreover, the induced SOX2 expression was completely abolished when TARBP2 was depleted in tamoxifen-treated MCF-7 cells (Figure 6F), which indicates that SOX2 accumulation is enhanced by tamoxifen-induced TARBP2. Paradoxically, we also noticed that the level of SOX2 mRNA remained upregulated in cells in which TARBP2 was knocked down (Figure S4), which suggests that TARBP2 may act post-transcriptionally to regulate SOX2 and act as a parallel mechanism for tamoxifen-mediated SOX2 induction. Taken together, these results suggested that SOX2 is the functional downstream target of the tamoxifen-TARBP2 axis, which modulates drug resistance. Following our findings that indicated the posttranscriptional regulation of SOX2, we further examined SOX2 protein stability in tamoxifen-treated MCF-7 (Figure 6G) and MCF-7/TR1 (Figure 6H) cells. Our results showed that SOX2 protein was degraded more slowly in tamoxifen-treated MCF-7 cells compared with untreated cells (Figure 6G) and in MCF-7/TR1 cells compared with parental MCF-7 cells (Figure 6H). Furthermore, the depletion of TARBP2 in tamoxifen-treated MCF-7 cells blocked the tamoxifen-stabilized SOX2 protein accumulation (Figure 6I). All these results suggest that SOX2 is a downstream factor that promotes tamoxifen resistance, which is regulated by tamoxifen-induced TARBP2 through posttranscriptional SOX2 protein stabilization.

### 3.6. Higher Expression of SOX2 Is Correlated with the Level of TARBP2 and Hormone Therapy Resistance in Breast Cancer Patients

**Figure 7:**
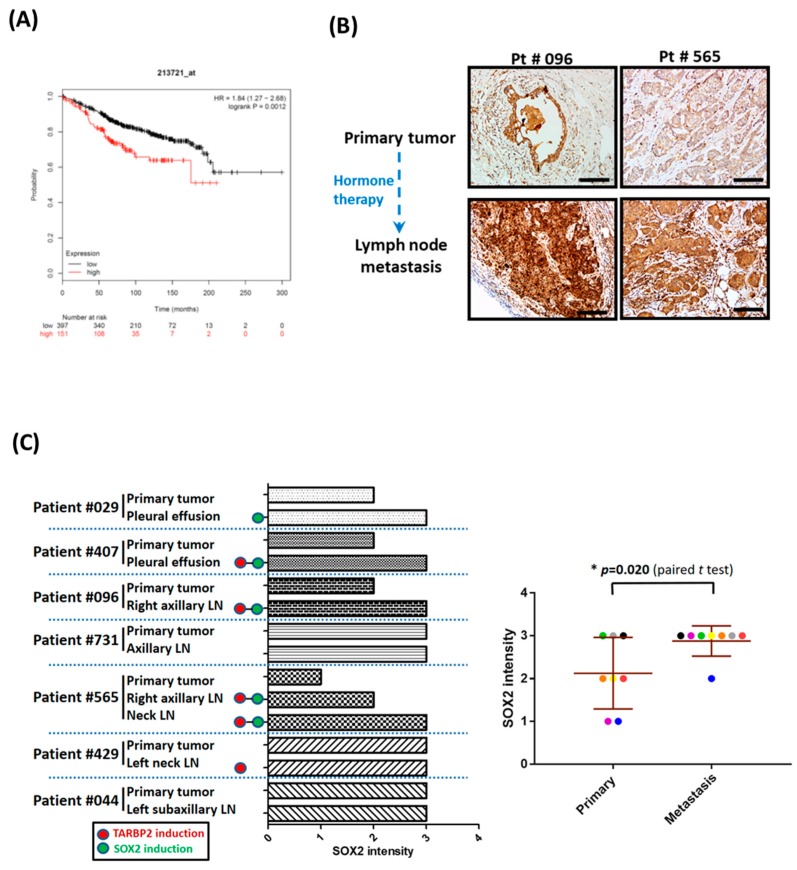

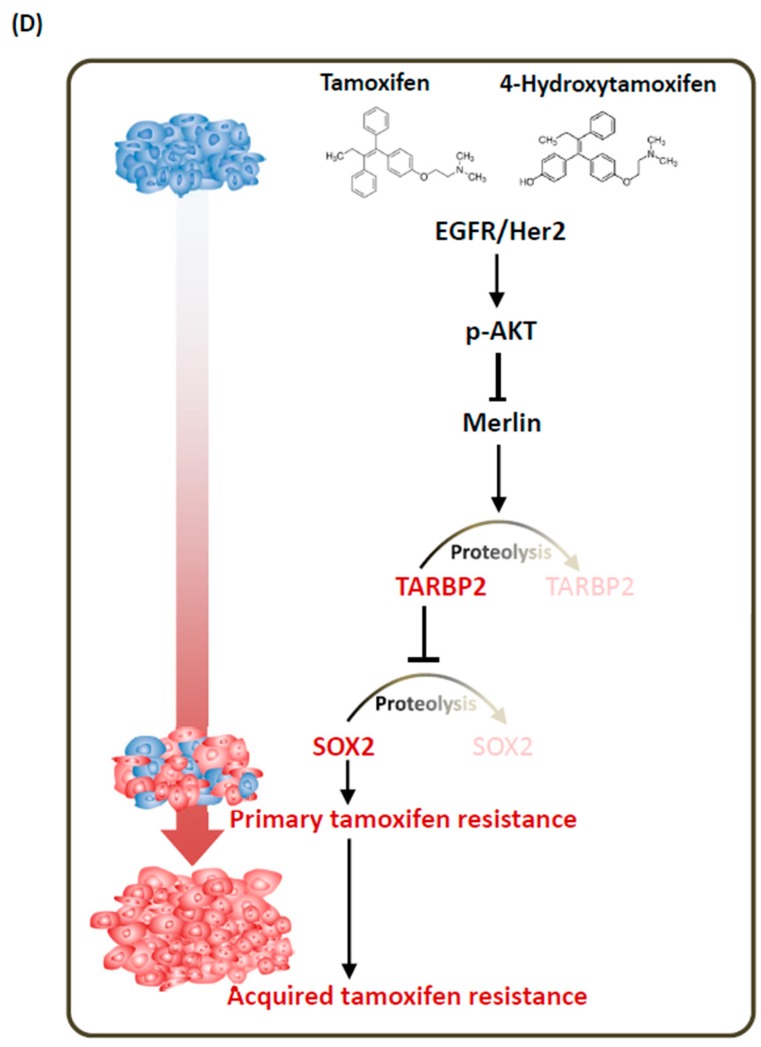
Both SOX2 and TARBP2 expression are elevated in hormone therapy-resistant tumor cells. (**A**) The correlation of SOX2 expression with the overall survival of ER-positive breast cancer patients was analyzed and downloaded using Kaplan-Meier Plotter (http://kmplot.com/). (**B**,**C**) Association of SOX2 expression and hormone therapy resistance in breast cancer tissues. Representative serial sections of Figure 1B showed images of SOX2 IHC in primary tumors and tumors in lymph nodes in cases of cancer recurrence (**B**). Scale Bar: 100 uM. Statistics of SOX2 protein expression levels in primary tumors and metastatic tumor cells in cases of cancer recurrence (**C**). (**D**) Resistance mechanism for tamoxifen–induced TARBP2-SOX2 in breast cancer.

In agreement with our findings that SOX2 is a downstream of TARBP2 that modulates tamoxifen resistance, we also observed that SOX2 expression is correlated with poor prognosis of ER+ breast cancer patients (Figure 7A). To ensure the expression pattern of SOX2 protein in hormone therapy-resistant breast cancer tissues, we determined SOX2 expression by IHC. In the paired tumor tissues, five metastatic lymph nodes or pleural effusions showed enhanced SOX2 expression compared with the corresponding primary tumors (Figure 7B,C), while four of the five tissue pairs showed elevated TARBP2 expression (Figure 7B,C and Figure 1B,C). These results support our in vitro findings that TARBP2 upregulates SOX2, which in turn induces tamoxifen resistance.

## 4. Discussion

The transcription factor SOX2 is an oncofetal protein that functions to maintain the pluripotency of embryonic stem (ES) cells and early organogenesis. A previous report has indicated that SOX2 is overexpressed in tamoxifen-resistant cells and that it confers stem cell-like and resistance phenotypes in breast cancer cells [31]. However, the exact upstream factor of SOX2, especially that which post-translationally regulates SOX2, is unclear. In this study, we showed that TARBP2 is a novel regulator of SOX2 under the condition of tamoxifen treatment. Several studies have reported on the degradation mechanisms of SOX2 protein. For example, phosphorylation of SOX2 by Akt was found to enhance protein stability [32]. In mouse ES cells, ubiquitin-conjugating enzyme E2S mediates K11-linked polyubiquitin chain formation of Sox2 protein for proteasome-mediated degradation [33]. Moreover, the Sox2 protein level in ES cells is modulated by a balance between phosphorylation and methylation, which is controlled by the interplay of Set7 and Akt [34]. These pathways may provide possible directions for further investigation of the detailed mechanism. In addition, we also noticed that two distinct pathways are responsible for tamoxifen-induced SOX2 expression. One pathway is the TARBP2-mediated protein stabilization, as mentioned above, while the other is the TARBP2-independent upregulation of SOX2 mRNA expression (Figure S4). Future studies on tamoxifen-induced SOX2 mRNA expression should also be considered.

As a tumor suppressor, Merlin is frequently inactivated in tumors of the nervous system [35,36,37,38,39,40,41]. As part of a complex with the Ezrin-Radixin-Moesin proteins, Merlin regulates contact inhibition and suppresses cellular invasive ability [42,43]. At the molecular level, several targets are controlled by Merlin, such as Ras and Rac, FAK, cyclin D1, and Pak1 [43,44,45,46,47,48]. It has long been known that Merlin interacts with TARBP2 and inhibits cell proliferation, anchorage-independent cell growth, and tumorigenesis in NIH3T3 cells [24]. The molecular mechanism that underlies these processes involves the ubiquitination and proteasomal degradation of TARBP2 [25]. In this study, we first revealed the link between tamoxifen and the Merlin-TARBP2-SOX2 axis and functionally connected this pathway to tamoxifen resistance of breast cancer cells (Figure 7D). In the future, it would be interesting to determine whether this pathway also regulates resistance to other anti-cancer drugs and if this phenomenon is observed in other types of human cancers.

Drug resistance is the bottleneck for cancer therapy. Clinical scenarios of drug resistance result from diverse mechanisms, and the early response to drug treatment for cancer depends on primary (de novo) resistance derived from the natural defensive ability of tumor cells. However, during treatment, cancer cells undergo clonal adaption, selection, and expansion into tumors with acquired resistance, which may also contribute to recurrence. Identification of the mechanism through which cells are resistant to tamoxifen is important since it is the most widely used endocrine therapy for women with breast cancer. In this study, we identified a novel mechanism of tamoxifen resistance in ER+ breast cancer cells through stabilization of TARBP2 protein and upregulation of SOX2. This pathway contributes not only to acquired resistance but also to de novo resistance. Interestingly, the induction of TARBP2 is triggered by tamoxifen, which suggests an unexpected effect of tamoxifen during hormone therapy. This study therefore revealed a missing link between the tamoxifen-induced signaling network and tamoxifen resistance, which provides important information for the design of better therapeutic approaches. However, there are several potential study limitations. First, it is unclear whether tamoxifen-induced TARBP2 contributes to cancer metastasis. Second, the expression of TARBP2 is theoretically intracellular and may only be detected by IHC. Therefore, further study on the possibility of extracellular TARBP2 will provide future directions for diagnosis, or even therapeutic strategies targeting TARBP2.

## 5. Conclusions

This study uncovered the oncogenic function of TARBP2 induced by tamoxifen treatment. Such a therapy-induced resistance mechanism demonstrates the link between the drug-induced molecular change of cancer cell and the resulting drug resistance. Overall, these results may inspire the scientific community to reconsider better strategies for tamoxifen use.

## Supplementary Materials

The following are available online at https://www.mdpi.com/2072-6694/11/2/210/s1, Figure S1: Establishment of tamoxifen-resistant cells, Figure S2: Overexpression of TARBP2 and its prognostic value in human breast cancer, Figure S3: Reduced ubiquitination of TARBP2 in tamoxifen-treated and resistant cells, Figure S4: Role of TARBP2 in the regulation of SOX2 mRNA expression.
