# Supplementary material for: TARBP2-Enhanced Resistance during Tamoxifen Treatment in Breast Cancer
Source: Cancers (Basel). 2019 Feb 12;11(2):210. doi: 10.3390/cancers11020210 (PMC6406945; doi:10.3390/cancers11020210)
Supplement: Supplementary file 1 [file cancers-11-00210-s001.pdf]

Supplementary Materials: TARBP2-Enhanced Resistance During Tamoxifen Treatment in Breast Cancer

Ming-Yang Wang, Hsin-Yi Huang, Yao-Lung Kuo, Chiao Lo, Hung-Yu Sun, Yu-Jhen Lyu, Bo-Rong Chen, Jie-Ning Li and Pai-Sheng Chen

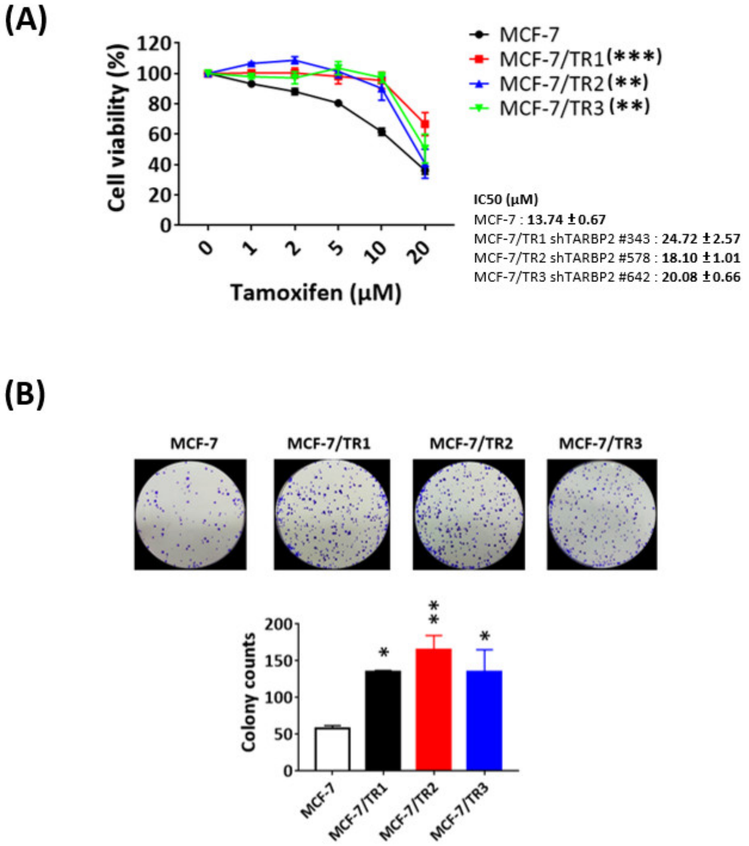

Figure S1. Establishment of tamoxifen-resistant cells. (**A**) Tamoxifen sensitivity of MCF-7/TR1, MCF-7/TR2 and MCF-7/TR3. Cells were treated with different concentrations of tamoxifen (1, 2, 5, 10, 20 μM) for 72 h, and cell proliferation was determined by MTT assay. (**B**) Colony-forming ability of MCF-7 and MCF-7/TR1, TR2, TR3 cells in the presence of tamoxifen. Cells were incubated with 5 μM tamoxifen for 7–10 days, the colony numbers of each group were counted by Image J analysis software. The experiments were repeated at least 3 times, * $p < 0.05$, ** $p < 0.01$ by one-way ANOVA.

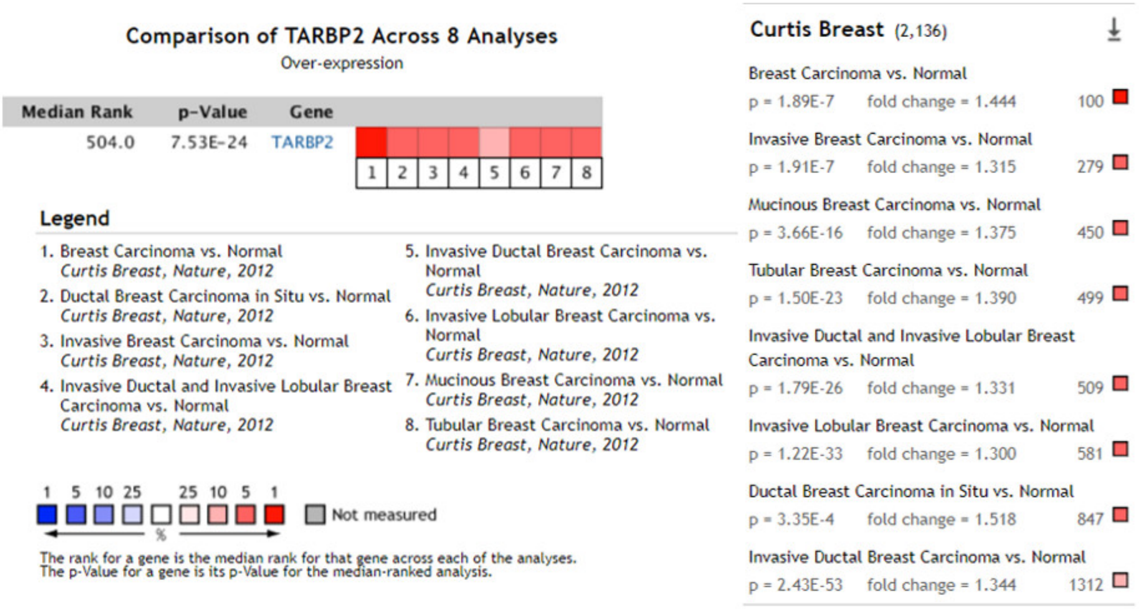
Comparison of TARBP2 Across 8 Analyses
Over-expression
Median Rank
p-Value
Gene
504.0
7.53E-24
TARBP2
1 2 3 4 5 6 7 8
Legend
1. Breast Carcinoma vs. Normal Curtis Breast, Nature, 2012
2. Ductal Breast Carcinoma in Situ vs. Normal Curtis Breast, Nature, 2012
3. Invasive Breast Carcinoma vs. Normal Curtis Breast, Nature, 2012
4. Invasive Ductal and Invasive Lobular Breast Carcinoma vs. Normal Curtis Breast, Nature, 2012
5. Invasive Ductal Breast Carcinoma vs. Normal Curtis Breast, Nature, 2012
6. Invasive Lobular Breast Carcinoma vs. Normal Curtis Breast, Nature, 2012
7. Mucinous Breast Carcinoma vs. Normal Curtis Breast, Nature, 2012
8. Tubular Breast Carcinoma vs. Normal Curtis Breast, Nature, 2012
1 5 10 25 25 10 5 1
%
Not measured
The rank for a gene is the median rank for that gene across each of the analyses.
The p-Value for a gene is its p-Value for the median-ranked analysis.
Curtis Breast (2,136)
Breast Carcinoma vs. Normal
p = 1.89E-7 fold change = 1.444 100
Invasive Breast Carcinoma vs. Normal
p = 1.91E-7 fold change = 1.315 279
Mucinous Breast Carcinoma vs. Normal
p = 3.66E-16 fold change = 1.375 450
Tubular Breast Carcinoma vs. Normal
p = 1.50E-23 fold change = 1.390 499
Invasive Ductal and Invasive Lobular Breast Carcinoma vs. Normal
p = 1.79E-26 fold change = 1.331 509
Invasive Lobular Breast Carcinoma vs. Normal
p = 1.22E-33 fold change = 1.300 581
Ductal Breast Carcinoma in Situ vs. Normal
p = 3.35E-4 fold change = 1.518 847
Invasive Ductal Breast Carcinoma vs. Normal
p = 2.43E-53 fold change = 1.344 1312

(**A**)

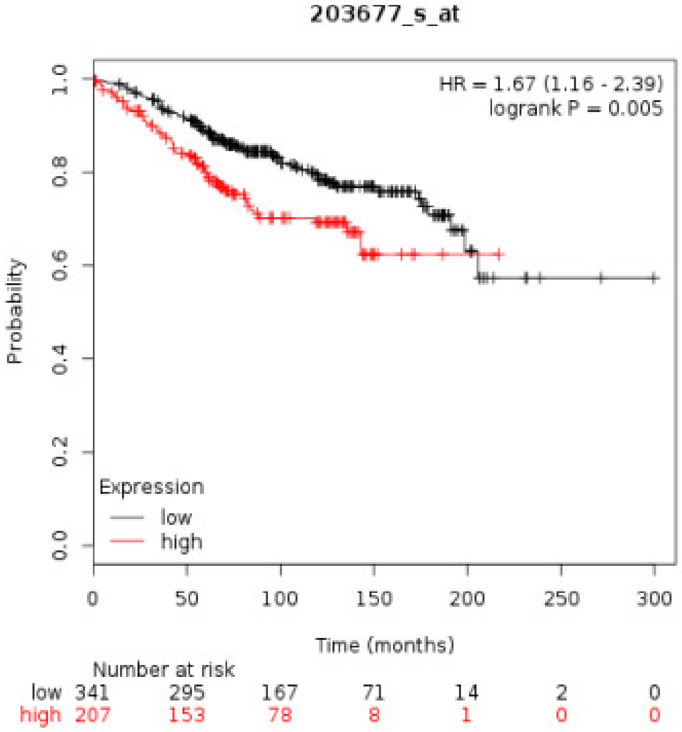
203677_s_at
HR = 1.67 (1.16 - 2.39)
logrank P = 0.005
Probability
0.0
0.2
0.4
0.6
0.8
1.0
Expression
low
high
0 50 100 150 200 250 300
Time (months)
Number at risk
low 341 295 167 71 14 2 0
high 207 153 78 8 1 0 0

(**B**)

DATA POSTPROCESSING	None
PROBE_NAME	9840 [MLRG Human 21K V12.0]
PROBE_DESCRIPTION	TAR (HIV) RNA binding protein 2
GENE_SYMBOL	TARBP2
GENE_DESCRIPTION	TAR (HIV-1) RNA binding protein 2
DATASET	GSE9893
CANCER_TYPE	Breast cancer
SUBTYPE	
N	155
ENDPOINT	Overall Survival
PERIOD	Months
COHORT	Montpellier, Bordeaux, Turin (1989-2001)
ARRAY TYPE	MLRG Human 21K V12.0
CONTRIBUTOR	Chanrion
ADJUVANT THERAPY	Tamoxifen: 100%
ADJUVANT THERAPY	Radio: 85%
ER STATUS	Positive: 95%
NEOADJUVANT THERAPY	NONE
SAMPLE TYPE	Frozen
CUTPOINT	0.81
MINIMUM *P*-VALUE	0.000643
CORRECTED *P*-VALUE	0.017549
$\ln(HR_{high} / HR_{low})$	0.96
COX *P*-VALUE	0.014213
ln(HR)	0.44
HR [95% CI]	1.55 [1.09 - 2.19]

(**C**)

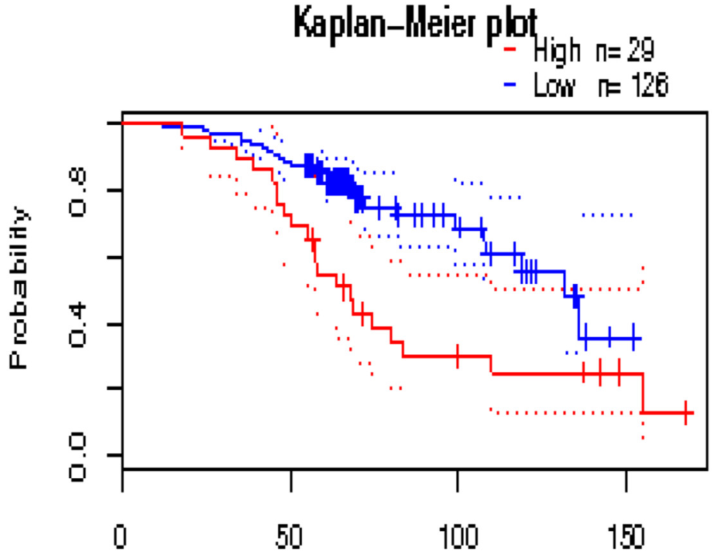

(**D**)

Figure S2. Overexpression of TARBP2 and its prognostic value in human breast cancer. (**A**) The expression of TARBP2 was analyzed and downloaded using Oncomine (www.oncomine.org). Relative expression of TARBP2 in different subtypes of breast cancer were analyzed for comparison with normal tissue. (**B**) The correlation of TARBP2 expression with overall survival in ER-positive breast cancer patients was analyzed and downloaded using Kaplan-Meier Plotter (http://kmplot.com/). (**C**) The correlation of TARBP2 expression with the prognosis of breast cancer patients was analyzed and downloaded using PrognoScan (http://www.abren.net/PrognoScan).

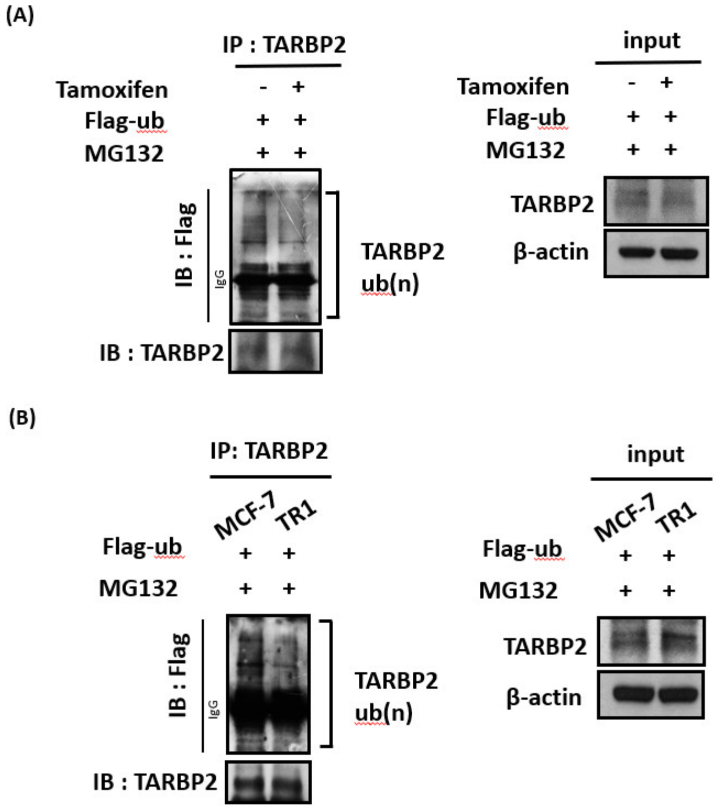

Figure S3. Reduced ubiquitination of TARBP2 in tamoxifen-treated and resistant cells. Flag-ubiquitin was expressed in MCF-7 (**A**,**B**) and MCF-7/TR1 cells (**B**) for TARBP2 ubiquitination assays. The immunoprecipitates isolated by anti-TARBP2 antibodies were subjected to western blot analysis for determining the expression of Flag-ubiquitin, indicating the ubiquitination levels of TARBP2. Cells were treated with MG132 to enhance the accumulation of poly-ubiquitinated TARBP2.

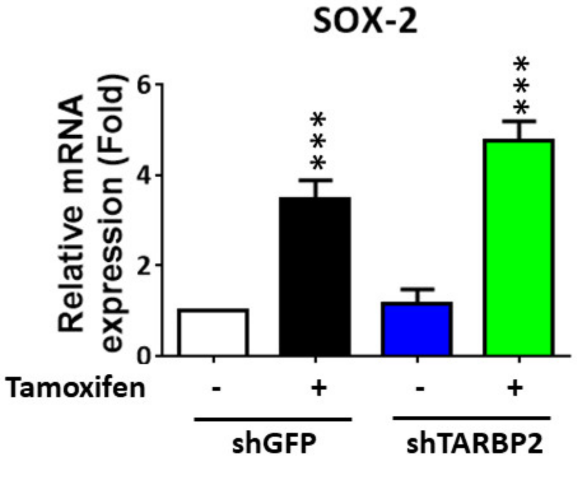

Figure S4. Role of TARBP2 in the regulation of SOX2 mRNA expression. RNA was isolated from cells as indicated in Figure 6F to analyze the mRNA expression of SOX2 by reverse-transcription PCR (qRT-PCR). The experiments were repeated at least 3 times, * $p < 0.05$, ** $p < 0.01$ by *t*-test.

© 2019 by the authors. Submitted for possible open access publication under the terms and conditions of the Creative Commons Attribution (CC BY) license (http://creativecommons.org/licenses/by/4.0/).
